# Supplementary material for: Preoperative Anxiety in Patients with Pancreatic Cancer: What Contributes to Anxiety Levels in Patients Waiting for Surgical Intervention
Source: Healthcare (Basel). 2023 Jul 17;11(14):2039. doi: 10.3390/healthcare11142039 (PMC10380009; doi:10.3390/healthcare11142039)

**SUPPLEMENTARY MATERIAL**

**Figure S1. Flow chart describing the whole population attending the Pancreas Institute of the University Hospital of Verona between June 2017 and June 2018 (target period of the study) on the basis of intervention type (source: administrative data provided by the Pancreas Institute). The first part of this flow chart was already reported in Del Piccolo et al. 2021. Here we added the final selection of the study sample included in this paper.**

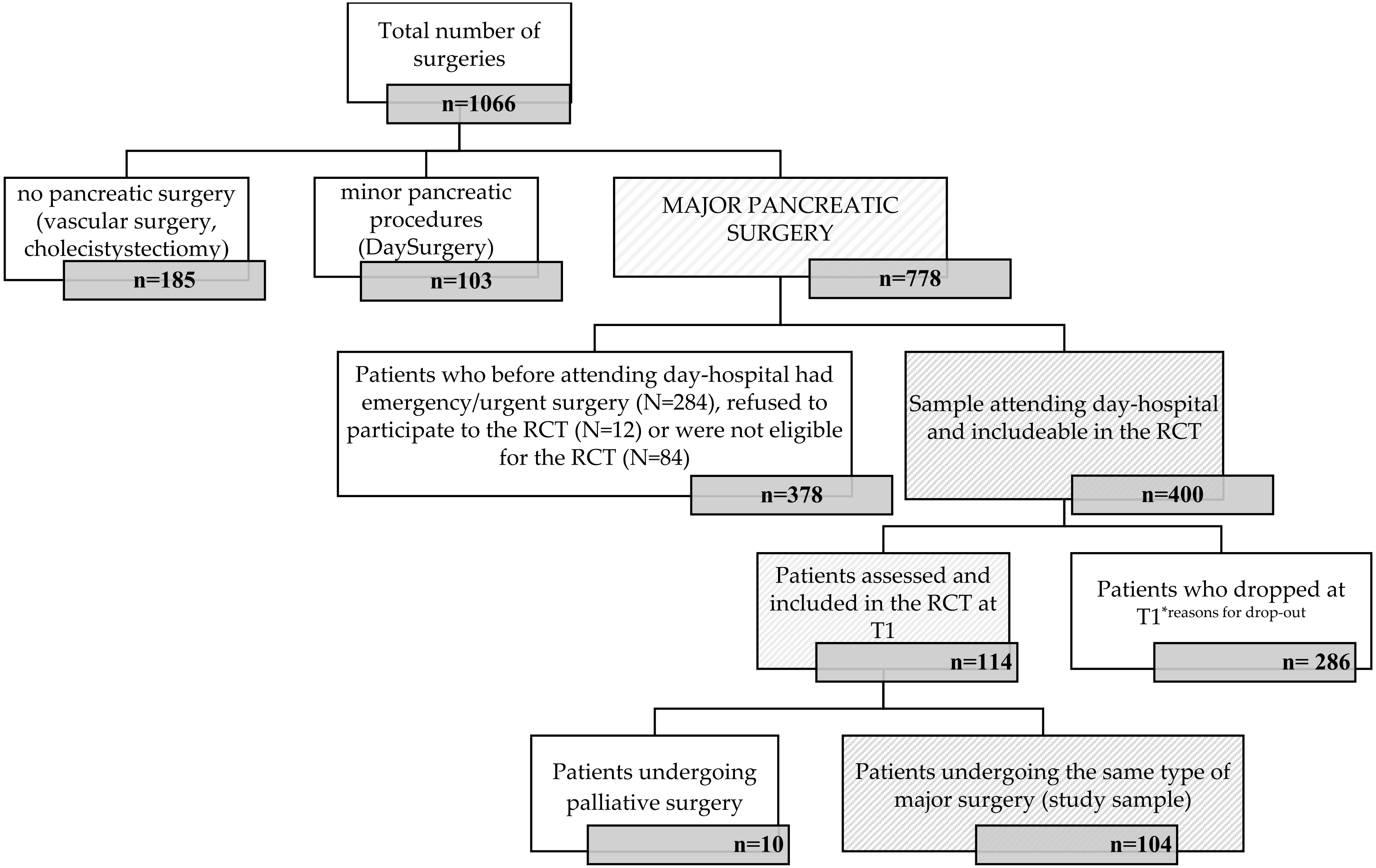

| *REASONS FOR DROP-OUT | Total: N=286 (100%) |
|---|---|
| Hospitalization after 5 p.m. when the clinical psychologist that received patients was absent | **72 (25.2%)** |
| Hospitalization during festivity days: weekends or holidays when the clinical psychologist was absent | **68 (23.8%)** |
| Hospitalization when the clinical psychologist was not available having other clinical duties | **36 (12.6%)** |
| Changes in surgery planning (hospitalization the same day of surgery, surgery postponed or moved in a different hospital) | **9 (3.1%)** |
| Patient not available (for clinical reasons or because attending other clinical examinations) | **14 (4.9%)** |
| Patient did not undergo surgery | **76 (26.6%)** |
| Included by mistake (not satisfying inclusion criteria) | **8 (2.8%)** |
| Patient declined to participate | **3 (1.0%)** |

**Table S1**. Frequency distribution (%) of STAI-S items (n=104)

| Items | | | (1) | (2) | (3) | (4) |
|---|---|---|---|---|---|---|
| 1. I feel calm* | | | 15 | 19 | 51 | 15 |
| 2. I feel secure* | | | 12 | 18 | 49 | 21 |
| 3. I am tense | | | 41 | 30 | 11 | 18 |
| 4. I feel strained | | | 66 | 16 | 6 | 12 |
| 5. I feel at ease* | | | 22 | 25 | 36 | 17 |
| 6. I feel upset | | | 72 | 14 | 8 | 6 |
| 7. I am presently worrying over possible misfortunes | | | 30 | 29 | 25 | 16 |
| 8. I feel satisfied* | | | 14 | 24 | 32 | 30 |
| 9. I feel frightened | | | 52 | 28 | 10 | 10 |
| 10. I feel comfortable* | | | 23 | 24 | 31 | 22 |
| 11. I feel self-confident* | | | 10 | 14 | 38 | 38 |
| 12. I feel nervous | | | 50 | 28 | 11 | 11 |
| 13. I am jittery | | | 45 | 29 | 10 | 16 |
| 14. I feel indecisive | | | 84 | 6 | 7 | 3 |
| 15. I am relaxed* | | | 38 | 20 | 36 | 6 |
| 16. I feel content* | | | 49 | 15 | 18 | 18 |
| 17. I feel worried | | | 24 | 28 | 18 | 30 |
| 18. I feel confused | | | 79 | 9 | 5 | 8 |
| 19. I feel steady* | | | 44 | 27 | 24 | 5 |
| 20. I feel pleasant* | | | 13 | 25 | 47 | 15 |
| | mean | sd | range | Kurtosis | Skewness | alpha |
| Total score | 43.27 | 12.92 | 20-77 | 2.7 | 0.6 | 0.93 |

(1)= not at all; (2) = somewhat; (3) = moderately; (4) = very much. * Items to reverse in the score calculation

**Table S2**. Frequency distribution (%) of APAIS items (n=104)

| Items | (1) | (2) | (3) | (4) | (5) | (6) |
|---|---|---|---|---|---|---|
| 1. I am worried about the anaesthetic | 47 | 18 | 10 | 9 | 7 | 9 |
| 2. the anaesthetic is on my mind continually | 61 | 17 | 7 | 1 | 6 | 8 |
| 3. I would like to know AMAP about the anaesthetic | 70 | 15 | 8 | 1 | 3 | 3 |
| 4. I am worried about the procedure | 13 | 13 | 21 | 11 | 22 | 20 |
| 5. the procedure is on my mind continually | 20 | 19 | 19 | 7 | 14 | 21 |
| 6. I would like to know AMAP possible about the procedure | 56 | 19 | 13 | 5 | 3 | 4 |
| **Scales** | **Mean (sd)** | **range** | **Kurtosis** | **Skewness** | | **alpha** |
| APAIS total score | 15.0 (6.2) | 6-31 | | | | 0.73 |
| APAIS anaesthesia | 4.4 (3.1) | 2-12 | 3.6 | 1.3 | | 0.89 |
| APAIS surgery | 7.1 (3.3) | 1-12 | 1.8 | <0.01 | | 0.85 |
| APAIS info | 3.5 (2.1) | 2-12 | 5.5 | 1.6 | | 0.52 |

Likert score ranges from (1)= not at all to (6) = extremely

**Table S3**. Pearson Correlation between variables included in the model (n=104)

| rho | STAI-S | PHQ9 | STAI-T | APAIS-S | APAIS-A |
|---|---|---|---|---|---|
| PHQ9 | 0.41 | | | | |
| STAI-T | 0.55 | 0.40 | | | |
| APAIS-S | 0.30 | 0.31 | 0.30 | | |
| APAIS-A | 0.61 | 0.26 | 0.38 | 0.25 | |
| APAIS-I | 0.25 | 0.23 | 0.33 | 0.36 | 0.22 |

**Figure S2: Hyothesis inderlined in the two models compared in hour study.**

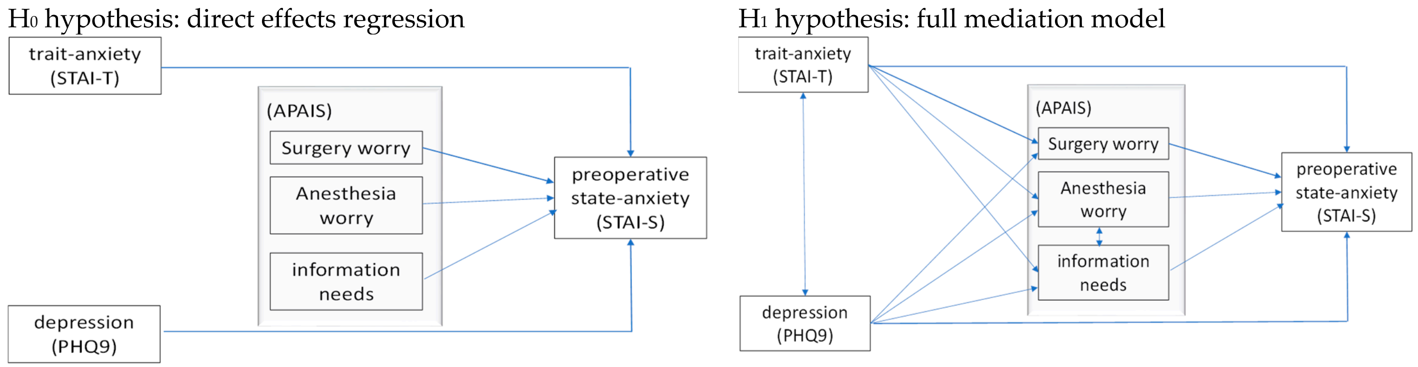

# Figure S3: A-posteriori power calculation

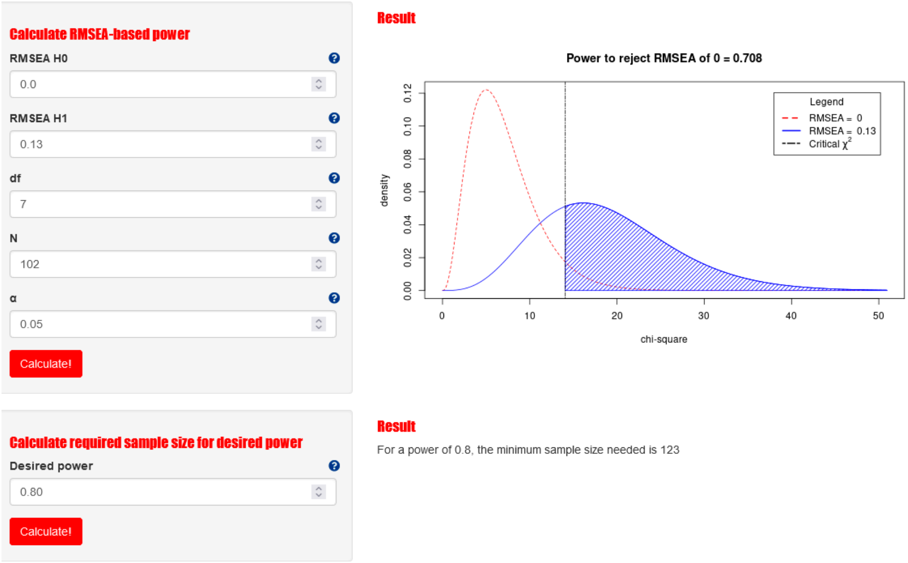

Supplement: Supplementary file 1 [file healthcare-11-02039-s001.zip › healthcare-2320571-supplementary.pdf]
